# The “Stars and Stripes” Metaphor for Animal Regeneration-Elucidating Two Fundamental Strategies along a Continuum

**DOI:** 10.3390/cells2010001

**Published:** 2012-12-27

**Authors:** Baruch Rinkevich, Yuval Rinkevich

**Affiliations:** 1 Israel Oceanography and Limnological Research, National Institute of Oceanography, PO Box 8030, Haifa 31080, Israel; 2 Stanford Institute for Stem Cell Biology and Regenerative Medicine, Stanford University School of Medicine, Stanford 94305-5323, CA, USA; E-Mail: ryuval@stanford.edu

**Keywords:** asexual reproduction, blastema, botryllid ascidians, digit tip, epimorphosis, morphallaxis, somatic embryogenesis, regeneration, whole body

## Abstract

A number of challenges have hindered the development of a unified theory for metazoan regeneration. To describe the full range of complex regeneration phenomena in Animalia, we suggest that metazoans that regenerate missing body parts exhibit biological attributes that are tailored along a morpho-spatial regeneration continuum, illustrated in its polar scenarios by the USA “stars and stripes” flag. Type 1 organisms (“T1, ‘stars’”) are typical colonial organisms (but contain unitary taxa) that are able to regenerate “whole new stars”, namely, whole bodies and colonial modules, through systemic induction and sometimes multiple regeneration foci (hollow regeneration spheres, resembling the blastula) that compete for dominance. They regenerate soma and germ constituents with pluripotent adult stem cells and exhibit somatic-embryogenesis mode of ontogeny. Type 2 organisms (“T2, ‘stripes’”) are capable of limited regeneration of somatic constituents via fate-restricted stem cells, and regenerate through centralized inductions that lead to a single regeneration front. T2 organisms are unitary and use preformistic mode of ontogeny. T1 and T2 organisms also differ in interpretation of what constitutes positional information. T2 organisms also execute alternative, less effective, regeneration designs (*i.e.*, scar formation). We assigned 15 characteristics that distinguish between T1/T2 strategies: those involving specific regeneration features and those operating on biological features at the whole-organism level. Two model organisms are discussed, representing the two strategies of T1/T2 along the regeneration continuum, the *Botrylloides* whole body regeneration (T1) and the mouse digit-tip regeneration (T2) phenomena. The above working hypothesis also postulates that regeneration is a primeval attribute of metazoans. As specified, the “stars and stripes” paradigm allows various combinations of the biological features assigned to T1 and T2 regeneration strategies. It does not consider any concentration gradient or thresholds and does not refer to the “epimorphosis” and “morphallaxis” terms, regeneration types across phyla or across body plans. The “stars and stripes” paradigm also ignores, at this stage of analysis, cases of regeneration loss that may obscure biological trajectories. The main advantage of the “stars and stripes” paradigm is that it allows us to compare T1/T2 regeneration, as well as other modes of regeneration, through critical determining characteristics.

## 1. Introduction

Regeneration of damaged tissues, lost parts and whole bodies is a basic biological phenomenon, an integral feature of multicellular organisms. It involves a complex temporal and spatial interplay of molecular signaling cascades, cell divisions, motion and interpretation of chemical and mechanical stimuli. Cross-phyla studies have indicated that regeneration, while variable at most levels of biological design, portrays extensive conservation of developmental signaling pathways [1]. However, the nature of signals that instigate the process of regeneration and the trail of induced effector cells remains elusive, as various regeneration scenarios are presumably fashioned by different cellular sources and mechanisms, including dedifferentiated elements and dedicated stem cells [1,2]. As an illustration, in planarians regeneration is carried out by adult stem cells, including pluripotent stem cells as building blocks, whereas in vertebrates an assortment of lineage-restricted progenitor cells regenerate various tissues (reviewed in [2]). Even a cursory assessment of regenerative phenomena in multicellular organisms reveals marked variation [1,2,3,4,5]. Moreover, while metazoans have evolved a rich array of regeneration strategies, they vary widely in their ability to recover from major morphological losses. Proper regeneration of tissues/organs requires the collaboration of: (a) the cellular building blocks, including stem cells [6]; (b) developing vascular network that meets nutrient and oxygen demands; (c) an extracellular matrix that creates the adhesive milieu for tissue/organ shapes [7]; and (d) positional information that dictates the correct shape of the regenerating organ [8,9]. 

## 2. In Rerum Natura

Of the wide range of vertebrate taxa that are capable of re-growing amputated organs and body parts, the most notable cases are displayed by amphibian limbs, lens and retina [5,10] and a few organs in mammals, including deer antlers, terminal phalanges of humans, marsupials and rodents, liver in mice and humans, and ear tissues in certain strains of mice and rabbits [3,11,12,13]. Ample documented cases present a wide range of regeneration capacities in invertebrate taxa. A phylogenetic perspective that considers all available data reveals a decrease in regenerative abilities concomitant with an increase in animal body and tissue complexity [4,8]. Further supportive comes from the wide scope of rehabilitative outcomes, including whole body regeneration (WBR) capacities, recorded in less complex multicellular organisms such as sponges [14], cnidarians [15] and flatworms [16]. The WBR competency is lost in higher vertebrates. However, some vertebrates, such as salamanders, frogs and fish, while unable to regenerate the entire body, can regrow partial or complete tissues and organs. An exception to this phylogenetic trait is the WBR phenomenon in the urochordate subfamily Botryllinae. In this group of sedentary colonial organisms, a fully functional adult regenerates within <2 w from isolated minute fragments (0.1 mm apiece) of blood vessels, each containing only 100–300 blood cells [1,17,18,19,20,21,22]. 

Following the same comparative line, while many sponges, cnidarians, platyhelminths, echinoderms and tunicates are capable of regenerating almost any body structure, other closely related sister taxa are incapable of regeneration, or exhibit a diminished capacity (e.g., [14,23]).

Substantial research efforts have been devoted to elucidating the sources of new cell types in regeneration, identifying molecular interactions and signaling molecules required for proper regeneration (reviewed in [1,2,24]) and clarifying 3D structural rules. Responses to injury on the morphological level usually start with processes targeting wound healing, proceeded by accumulation of cells to be used as building blocks in regeneration, and culminating in pattern formation of regenerated structures [25,26]. The emerging studies on common routes and evolutionary basis of regeneration have primarily focused on the molecular and cellular events, providing neither a new comprehensive understanding of regeneration, nor any novel testable metaphor for regeneration.

## 3. The Blastema—Res Ipsa Loquitur

The blastema is a transient structure appearing during regeneration of some vertebrate/invertebrate taxa and associated with localized and time-limited proliferation of progenitor cells. At the level of histology, the blastema is a mass of seemingly homogeneous, undifferentiated progenitor cells, capable of regeneration organs or body parts. Some invertebrates (such as planarians; [2]), amphibians and certain species of fish can produce a blastema during either juvenile or adult stages. The regeneration blastema resembles, in many ways, the progress zone of the developing limbs whereas the dorsal-ventral and anterior-posterior axes between the stump and the regenerating tissue are conserved. Therefore, the blastema becomes apparent as an autonomous regeneration entity, a self-organizing system over variable linear dimensions. In the chordates, the blastema consists of mesenchymal cells covered by a layer of epidermis called the apical epithelial cap, which plays a vital role in regeneration. Fibroblasts from the connective tissue migrate across the amputation surface and multiply to form a blastema, continue to proliferate, and eventually re-differentiate to regenerate missing organs (e.g., [27,28]). Thus, in contrast to the common wound healing process in mammals, no scar is formed. The blastema cells are autonomous, “memorizing” their original position, even when the cells are placed in a different positional environment [28]. More interesting is the finding that seemingly dedifferentiated blastema cells in axolotl limb regeneration (e.g., cartilage, muscle, and neuronal precursors) remain within their original lineage [29]. Most animals, however, do not produce blastemas.

It is particularly striking that most studies on blastemas in vertebrates and invertebrates group all “blastemas” into a single entity, without differentiating between potentially distinct blastema types.

## 4. The Theoretical Concept for Regeneration—Does It Hold?

About 110 years ago, Thomas Hunt Morgan [26] coined the umbrella term “regeneration” to characterize the common features of the many diverse phenomena of body rehabilitations. He introduced a clear terminology for the study of regeneration and suggested two terms reflecting the major different regenerative processes, “epimorphosis” and “morphallaxis”. *Epimorphosis* refers to regenerative phenomena in which active cellular proliferation occurs prior to the replacement of the lost body part, and is frequently characterized by the formation of a “blastema” structure. This type of regeneration is frequently encountered in planarians, molluscs, echinoderms, urochordates and vertebrate limb/tail regeneration [4]. The cellular basis for this regeneration, which is controversial, contains dedifferentiation or transdifferentiation of differentiated cells and adult somatic stem cells [1,30]. *Morphallaxis* refers to the type of regeneration in which lost body parts are replaced by remodeling of the remaining tissue, with little or no cellular proliferation. A classic example of this is the fresh water organism *Hydra* but other organisms such as tunicates also display this mode of regeneration [31]. The concepts of epimorphosis/morphallaxis remain central paradigms in the field of regeneration, even though molecular evidence for a common ancestral mechanism that exhibits one or both of these cellular paradigms is presently lacking [4], despite new findings challenging their universal applicability and soundness. Furthermore, we now know that more than different modes of regeneration can operate in different tissues of the same organism or in different species within the same taxonomic group [4,30,31,32,33]. In the vertebrates, fibrosis, a mechanism distinct from epimorphosis/morphallaxis for injury-induced “repair,” patches and remodels a wound with scar tissue and lowers its functional capacity. Even tissues capable of regeneration may be repaired by fibrosis if their wound size exceeds their regenerative capacity [34].

Unlike normal development processes, regeneration is often triggered by unpredictable events and is derived from disorganized morphologies [3,17]. The central theoretical concept used in discussions on regeneration is the positional information scheme, suggested by Wolpert [35]. In Wolpert’s French flag metaphor [35], a concentration gradient is formed by the diffusion of a morphogen from a source and cells near this gradient respond to concentration thresholds. While being one of the most persuasive concepts in biological sciences, current research (e.g., [36,37,38,39]) has indicated that positional information is harder to define than originally envisioned by Wolpert [35]. Moreover, the exact nature of the French flag metaphor, *i.e.*, the injury stimulus that “alerts” the organism to the loss of- and the establishment of new positional information values, remains elusive. Thus far, Wolpert’s French flag metaphor, which has not provided a comprehensive understanding of regeneration [36] is being challenged by studies showing that critical patterning cues are positioned independently of a local organizer [37] or that blastemas may form away from a regeneration plane [30]. Furthermore, cell lineage analyses in salamanders, mice, *Xenopus* and zebrafish [40,41,42,43] have collectively demonstrated that stem cells in the vertebrate limb regeneration are predetermined and fate-restrict. This means that key cellular fates in regeneration are independently developing from organizer/morphogen gradients, falsifying Wolpert’s “French flag metaphor” for positional information. It is also suggested [38] that regeneration processes, like vertebrate limb-bud development, are controlled by a 4D patterning system integrating positive and negative regulatory feedback loops rather than thresholds set by morphogen gradients. However, while the molecular background underlying all regeneration processes is absolutely unclear, rules by which organs regenerate through the establishment of a new positional information can sometimes be formulated in an experimentally verifiable manner (with combined quantitative and qualitative values), even if self-organizing capabilities are present [39].

Significant strides have been made towards understanding the nature and evolutionary basis of regeneration in response to central question: Why certain taxa have reduced or lost altogether their regenerative powers in contrast to their regeneration-competent sister taxa. We understand even less when regeneration is discussed in conjunction with other developmental phenomena, such as asexual reproduction, embryogenesis, growth and even cancer [4,6,44,45,46]. This stems from the fact that regeneration processes in Animalia differ profoundly from each other, characterized by the presence or absence of blastema, the development of a single *vs*. multiple regeneration centers, centralized *vs*. systemic induction for regeneration [3], epimorphosis *vs*. morphallaxis, dedifferentiation of mature tissue *vs*. stem cells proliferation and various combinations of all above regeneration modes. Even the intensively studied model systems, amphibian limbs and tails, fish fins, planarians and hydra, although offering incredibly useful tools in regeneration research and revealing widely shared features, do not provide unifying principles of regeneration [30]. In addition, most regeneration studies involve manipulations of a single species in a non-comparative context.

## 5. The “Stars and Stripes” Paradigm for Animal Regeneration

**Figure 1:**
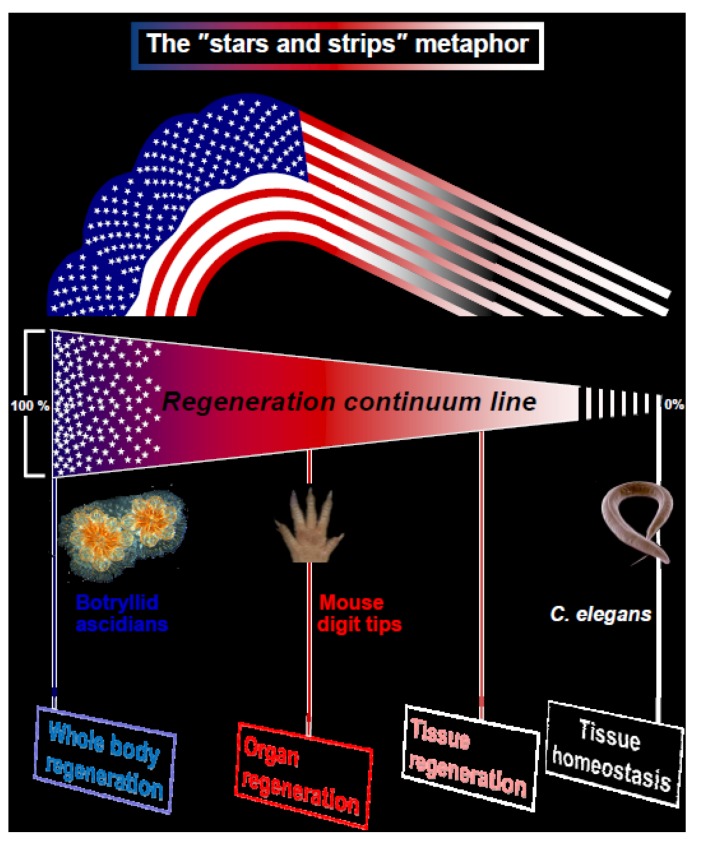
Regeneration in metazoans. The illustrated metaphor of the USA “stars and stripes” flag, with four assigned distinct regeneration turning points outlined along the multicellular organisms “regeneration continuum”. T1 and T2 organisms specify the “Whole body regeneration” and “Organ regeneration” strategies with model cases, the botryllid ascidians regeneration and the murine digit tip regeneration, respectively.

Here we suggest a new metaphor for regeneration, illustrated by the American Flag, the “stars and stripes” paradigm for animal regeneration (Figure 1). We assert that the different types of regeneration in metazoans can be outlined along a “regeneration continuum” with four distinct regeneration turning points, or basic strategies (WBR, organ regeneration, tissue regeneration, tissue homeostasis; Figure 1) embodying the regeneration competency up-stream with the "stars and stripes" strategies. Type 1 organisms (T1, the “stars” e.g., sponges, cnidarians, tunicates, bryozoans; exemplified here through Whole Body Regeneration in botryllid ascidians; Table 1) regenerate whole bodies (whole new stars) or modules, including soma and germ constituents, via systemic induction and regeneration foci (hollow regeneration spheres, resembling the blastula, a hollow sphere composed of a single pluripotent cell layer which is the strict invention of Animalia; [47]) that compete for dominance. It is interesting to note that T1 regeneration events in various organisms [17,48,49], as aberrant events such as early teratomas and EC foci, go through a stage that resembles the blastocyst in structure. Above events also express of the same stemness signatures in soma and germ lines. Thus, the conserved morphological features of these structures most likely underlie evolutionary sustained cellular attributes and molecular cascades. Type 2 (T2, the “stripes”; exemplified here through murine digit tip regeneration [DTR]; Table 1) organisms, which display locally-induced limited regeneration along axes, go through a single regenerating plane that is restricted to the soma and show disparate stemness signature types for soma and germ lines (Table 1). Both strategies differ in modes of ontogeny (somatic embryogenesis versus preformistic, respectively), in the types of stem cells that are used as the building blocks for regeneration (pluripotent stem cells versus germ-layer and lineage restricted stem cells; respectively), in the end products (soma and germ lineages versus distinct somatic lineages only) and in the existence/lack/*de novo* formation of pluripotent adult stem cells (Table 1). Botryllid ascidians are capable of WBR from minute vasculature fragments, whereas mice are capable of forelimb/hindlimb DTR when amputated distal to the last interphalangeal joint. DTR also shares similarities with limb regeneration in anural amphibians and regeneration of missing distal finger portions in children/adults; undertaking divergent routes from the ascidians WBR process. The inherent scope of the two regeneration modes is illustrated below by the T1/T2 strategies of the “stars and stripes” model, also showing different interpretation of what constitutes positional information (Table 1).

**Table 1:** Biological features assigned to the “stars and stripes” pole strategies of metazoans” regeneration, following the two model regeneration cases: the whole body regeneration (WBR) of botryllid ascidians (the allegory of “stars” in the American flag) and the murine digit tip regeneration (DTR) (the allegory of “strips” in the American flag). The following is based on [3,17,18,20,50,51,52,53,54,55]; and unpublished results).

No.	Biological feature	WBR of botryllid ascidians	Murine DTR
1	Magnitude of regeneration	Whole body	Repair of only amputated parts
2	Role played by discarded building blocks	center behind fragments restrict regeneration	Stump contains residual cells/tissues from missing part
3	Outcome tissues	Soma and germ constituents	Soma only
4	Cells of origin	Activation, mobilization and expansion of adult stem cells; also *de novo* emerged stem cells	Tissue resident; integrated expansions of lineage restricted stem cells from different embryonic germ layers
5	Type of regeneration induction	Systemic	Locally induced
6	Morphological features for regeneration	Signalling centres-foci (hubs), resembling blastocysts in structure	A regeneration plane
7	Number of regeneration units	Multiple hubs	Single front, morphologically homogeneous
8	Hierarchy	Regeneration foci compete for dominance; only a single hub/ fragment will regenerate	No hierarchy
9	Axes	Newly established order from morphological chaos	Directionality towards the periphery
10	Alternative regeneration pathways	None	“Repairing” through scar tissues
11	General morphological archetype	Colonial and unitary	Unitary
12	Competency for asexual reproduction	High	None
13	Mode of ontogeny	Somatic embryogenesis	Preformistic
14	Existence of totipotent adult stem cells	yes	no
15	Expression of the same stemness signatures in soma and germ line	yes	no

### 5.1. T1-The Complex WBR in Colonial Urochordates (Figure 2)

The colonial urochordate *Botrylloides leachi* is an encrusting colonial sea squirt, found in shallow waters along the Mediterranean Sea, under stones, on algae, pilings, floats and other substrata. Each colony is composed of up to several thousands of genetically identical modules, called zooids (each about 2–3 mm long) that are embedded within a gelatinous, semi-transparent tunic matrix. All zooids within a colony are arranged in parallel elongated and often serpentine rows, called systems, which are connected by a network of blood vessels from which pear-shaped vascular termini (ampullae) extend toward the colony margins. A *Botrylloides* colony grows by weekly and cyclical budding processes (each called blastogenesis) during which new zooids bud from the thoracic body wall of older zooids that simultaneously die at the end of each blastogenic cycle in a massive apoptotic event [21,50].

**Figure 2:**
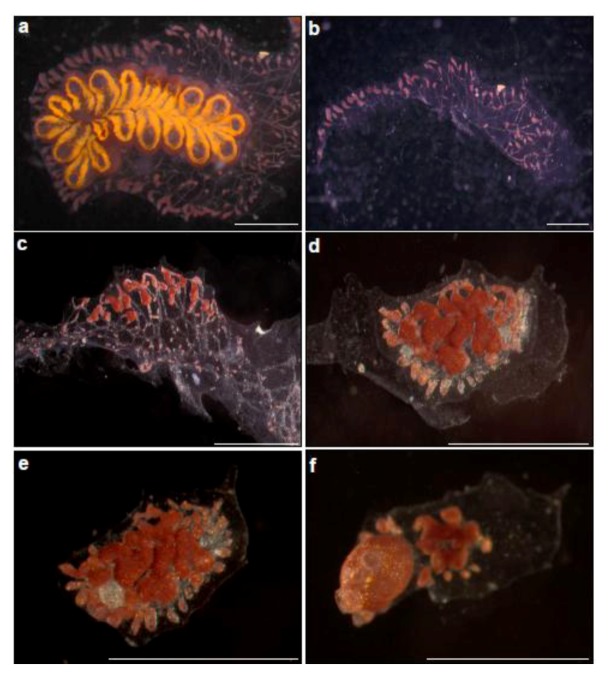
Whole body regeneration in *Botrylloides leachi* from the Mediterranean coast of Israel. (**a**) A colony growing on glass slide, just before zooids” dissection; (**b**) Immediately after zooids” dissection. A piece of the tunic containing marginal ampullae is left; (**c**–**f**) Whole body regeneration within two weeks. (**c**) Morphological changes, three days after dissection, the tunic fragment with ampullae constructions. This is follows by the formation of bud rudiment at the middle of the fragment (**d** = day 7), well developed bud (**e** = day 10) and the establishment of fully functional, filtering organism (the two extending siphons are seen), containing a single zooid (**f** = day 15).

WBR in *B. leachi* develops in vascular sites deprived of zooids, when a fully developed zooid regenerates from a miniscule blood vessel fragment within 10–14 days from initiation (containing approximately 100–200 blood cells). However, intact colonies or colonial fragments containing a single intact zooid, do not regenerate even if all blood vessels have been amputated [3,17,50]). This indicates that in addition to the local regenerative signals, there are systemic WBR signals that override any wound response. By observing meticulously early phases of WBR, Rinkevich *et al.* [3] have revealed the existence of discrete regeneration centers within vasculature lumens, haemocytes homing, proliferation and subsequent aggregations of haemocytes and blood cells within the regeneration foci. Further work [20] revealed that WBR develops through activation, mobilization and expansion of “dormant” cells, which normally line the internal vasculature epithelium of blood vessels. Following a mechanical insult, these cells expressed *de novo* the *Botrylloides Piwi* gene (*Bl-Piwi*), changed morphology and invaded niches of the vasculature lumen, where they proliferated and differentiated, regenerating a new whole functional organism. Mitomycin C treatments and siRNA knockdown of *Bl-Piwi* resulted in deficient cells, incapable of expanding or differentiating and in subsequent regeneration arrest. Interestingly, similar transient mobilization of *Piwi*^+^ cells recurs every week as part of colony blastogenesis, and during acute environmental stress, enabling the adaptation of colonial tunicates to the imposed developmental, physiological and environmental insults in the harsh shallow water marine environment [20]. These experiments indicate that circulating stem cells, most likely pluripotent in nature, are accountable for WBR in *B. leachi*.

While multiple buds usually appear simultaneously in newly established regeneration centers within vasculature fragments, this process always terminates in a single functional zooid per fragment. Retinoic acid (RA) was found to regulate diverse developmental aspects in WBR as the homologue of the RA receptor and a Retinaldehyde dehydrogenase (Raldh) related gene were expressed specifically in blood cells within regeneration centers and throughout bud development. The addition of RA inhibitors and RNAi knockdown experiments resulted in WBR arrest and bud malformations whereas the administration of all-trans RA to blood vessel fragments resulted in doubly accelerated regeneration and multi-bud formation, leading to restored colonies with multiple zooids [3]. This unconventional botryllid WBR system differs from other regeneration model systems in several fundamental traits, such as epimorphosis without blastema formation, induction of multiple restoration centers by circulating blood cells and concurrent restoration of both the entire soma and the germ line [3]. While several studies [18,19] have elucidated specific representations of candidate genes in WBR processes, including genes belonging to major signaling pathways, such as Notch/Delta, protein kinases, nuclear hormone receptors, GPCR and TGF-β signaling, and a broad conservation of immune signaling expressions in WBR, these results are not used in the discussion as we feel it is premature to discuss molecular pathways under the theme of the "stars and stripes" paradigm for animal regeneration.

### 5.2. T2-Regenerating the Mouse Distal Phalanx (DTR; Figure 3)

Several mammalian groups are able to regrow amputated forelimb and hindlimb digit tips through the distal interphalangeal joint, including newborn and adult mice, marsupials, and also human children and adults [51,56,57]. The distal tip of the digit is a morphologically complex organ derived from multiple and distinct embryonic origins and germ layers. Regeneration of the digit tips is controlled by the integrated regrowth of multiple tissues, culminating within a period of 2–3 months in a cosmetically and functionally normal looking digit.

When exploring the cellular source of mouse digit-tip regeneration, Rinkevich *et al.* [51] have documented that the regeneration was a cumulative effort of distinct stem cells and their daughter progenitors. Genetic fate mapping and clonal analysis of individual cells revealed that those stem cells were tissue resident and fate restricted (including the cells involved in angiogenesis), mimicking digit growth during development. Therefore, differences between organisms that progress through T1/T2 regeneration strategies may portray inherent differences in their embryonic development, *i.e.*, developmental fates of embryonic cells. While amputations that removed most structures of the distal digit have failed to regenerate, amputations that left residual bone, sweat glands and remnants of nail organs showed, after 70 days, partial to complete regrowth of distal structures, with histological indications of mesenchyme cells at the digit apex, and local cell proliferations within defined sites of the distal digit. Therefore, the presence of a “boundary” permissive to regeneration is another hallmark of T2 strategy, wherein successful regeneration takes place only if amputated parts are above a permissive stage of development or preformed up to a certain tissue boundary [40,41,51]. Transplantation of cyan-fluorescent-protein-expressing haematopoietic stem cells, and parabiosis experiments conducted between genetically marked mice confirmed that the stem/progenitor cells were tissue resident, not circulating as in WBR. A very similar regeneration strategy is observed following amputations of the salamander limbs, zebrafish fins and xenopus tail, collectively demonstrating that appendage regrowth in the vertebrates [29,42,43] is mediated by fate-restricted stem and progenitor cells that serve an evolutionarily conserved cellular mode for limb regeneration after amputation.

**Figure 3:**
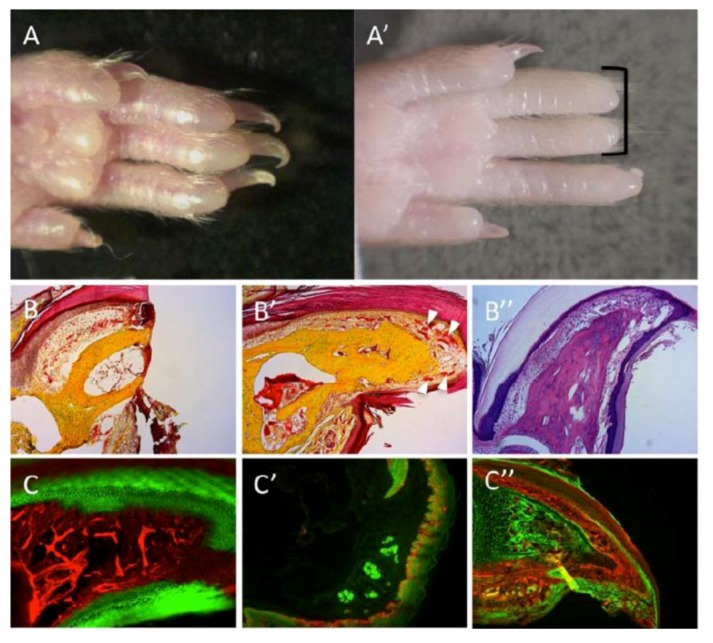
Regeneration of the mouse digit tip (DTR). (**A**) Hindlimb digits showing complete regrowth following 3-months from amputations. (**A’**) Absence of regrowth in digits amputated at more proximal planes (black bracket). (**B**,**B’**) Pentachrome staining; bone in yellow, endothelium and epidermis in red. (**B**) A section through the digit tips, two days following amputation. (**B’**) A section through a representative case for DTR, three months after amputation. Regrowth is visible within bone, nail, epidermis and vasculature (white arrowheads). (**B”**) Alcian-blue stain of a regenerated digit; bone and tendons are colored light purple, dermis is colored dark purple. (**C’**–**C”**) Genetic lineage tracing of tissues within the digit reveals germ-layer and lineage restriction throughout regeneration. *K14*^CreER^*R26*^mT/mG^ transgenic mice show lineage restriction of epidermis (GFP expression, **C**) during DTR; *EN1*^Cre^*R26*^mT/mG^ shows lineage restrictions of sweat glands and epidermis (GFP expression, **C’**); *Sox9*^Cre^*R26*^mT/mG^ shows lineage restriction of bone (GFP expression, **C”**).

Along with amphibian and mice appendage regeneration, enhanced expressions within the formed blastemas were recorded for MSX gene family members (e.g., MSX-1, MSX-2; [10,52,58]). In the mouse system, the expression pattern of MSX-1 within the regenerated digit tip correlates with the “permissive” boundary for regeneration [10], suggesting that MSX genes may have a role in the “dedifferentiation” or “stemness” of blastema cells [52,58]). However, recent genetic lineage tracing experiments have demonstrated that MSX-expressing cells are fate restricted to subsets of dermal cells within the dorsal and ventral digits and are by no means multipotent [53]. These results illustrate the idea of a “permissive” border of regrowth derived from tissue locations of the various fate-restricted progenitors, whereby amputations that remove most of the stem/progenitors would culminate in unsuccessful regrowth, which now lacks these necessary lineages. Such “borders” of regeneration (either spatial or temporal) may be less likely to occur in organisms that reserve cells with multipotent capacity.

### 5.3. Regeneration in Planaria-Setting within the T1/T2 Continuum

Flatworms, as representatives of the most basal triploblastic groups, exhibit diverse fission (architomy) and budding (paratomy) modes of asexual reproduction, exercising a wide-range of regeneration capacities, from WBR to cases where regeneration is lost [59]. This variation in regenerative powers is probably associated with the stem cells, called neoblasts, dispersed throughout flatworms” bodies, also representing the only dividing cells in asexual animals.

Considering the biological features assigned to the "stars and stripes" strategies for metazoansregeneration, model flatworms (e.g., *Planaria*) could be positioned downstream but close to the T1 pole of regeneration. Isolated minute planarian tissue fragments, containing about 10,000 cells (100–300 cells in *Botrylloides leachi*; [3,17,50]), may go through WBR, regenerating all somatic and germ cell types [60]. This trait, which is lost after whole body X-irradiation, is regained when the worm is partly shielded, even when the un-irradiated portion is away from amputated site, revealing motile stem cells; also somatic embryogenesis type of ontogeny (indicative of T1). This is further supported by experiments [61] demonstrating recruitment of neoblasts to tissue damaged sites following planarian amputation. Therefore, WBR in *Planaria* appears to be systemic (also supported by genes that are expressed systemically, in tissues away from the wound site and in multiple cell types [62]), indicative of T1 strategy. Neoblasts, one of the most prodigious stem cell types known, comprise 20% or more of the cells in an adult worm [63], which suggests that all neoblasts have clonogenic potential and that the apparent totipotency of neoblasts probably reflects the multifaceted products of multiple types of stem cells. Transplantations of individual neoblasts that have resulted in reconstituting multiple tissues within the planarian body further indicated that only some neoblasts are pluripotent [64]. Additionally, in contrast to the T1 model system, *Planaria* does not produce bud ramets as T1 organisms do but instead uses a regeneration plane, including the formation of a “blastema”, instead of regeneration centers as in *B. leachi*. This regeneration develops a single regeneration front, quite different from the multiple initiation centres in botryllid ascidians. In addition, there is no hierarchy during regeneration (indicative of T2 strategy) and there is an abridged repertoire of regenerating cell types or tissues/organs and reduced morphological complexity. Most important is the functional integration of newly emerged organs as compared with botryllid ascidians. The discussion above places *Planaria* regeneration downstream of *B. leachi* on the regeneration continuum, but far upstream of the murine digit tip regeneration.

## 6. Summary: Exitus Acta Probat

Our guiding working hypothesis is that regeneration is a primeval attribute of metazoans [65] that can be pretty confidently outlined and understood along a “regeneration continuum”, rather than a phenomenon which has evolved independently along evolution (multiphyletic emergence of regenerative phenomena) in response to a variety of biological circumstances or environmental settings. A number of challenges have hindered the development of a unified theory for metazoan’s regeneration. Most intriguing is the high degree of plasticity of the regeneration phenomena, reflected in distinct cellular processes (such as differentiation, dedifferentiation, transdifferentiation, *de novo* formation of stem cells; [2,3,12,15,30,33,51] and molecular cascades [3,18,19,20,23,40,51,52]. Consequently, private regeneration epiphenomena blur the overall picture, making the nature of these processes ambiguous. Furthermore, despite the plethora of data on regeneration processes in various organisms (and associated tenets), the literature raises questions on the various aspects of the regenerative proficiency in metazoans. As an example, while regeneration portrays extensive conservation of developmental signaling pathways through phyla [1] and cross-phyla studies reveal a decrease in regenerative abilities concomitant with increased animal body and tissue complexity [17], some taxa have restricted or altogether lost the regenerative capacity relative to their highly competent regenerating sister taxa (however, latent regeneration abilities that can be experimentally elicited may persist in lineages where regenerative ability is seemingly loss; [44]). Furthermore, regeneration processes vary not only between closely related taxa, but also within a single organism (still awaiting absolute confirmation). A good example is the basal chordate *Amphioxus,* which regenerates amputated tails through an epimorphic process, with active proliferation in an msx-expressing blastema [66], while oral cirri regenerate primarily through morphallaxis [67]. Another representative case [68] reveals discrepancies in regeneration ability in two sabellid worms (belonging to closely related genera) that employ different mechanisms to restore lost anterior body parts. Adults of one of these species deploy both morphallaxis and epimorphosis for bodily anterior end regeneration, whereas in the second species, only epimorphosis dominates regeneration. As understanding the above discrepancies remains rudimentary, for the clarity of the "stars and stripes" metaphor and the discussion, this opinion essay does not venture into the above territory.

Since regeneration processes are highly complex phenomena, work that aims to elucidate their nature should offer a cross-disciplinary view to reveal evolutionary roots. However, it should be noted that in a considerable number of higher taxonomic groups (even some entire phyla) there is almost no information on regenerative properties [23], and the records that are available deal with only a few taxa in a group. Still, comparative studies and novel metaphors for regeneration can suggest new lines of experimentation, not offered by any single model system. Understanding regeneration requires unifying hypotheses as explanations for complex regeneration programs are not obviously intuitive. Here, we suggest a testable novel metaphor for regeneration, illustrated by the American Flag, the "stars and stripes". This metaphor is exemplified through two disparate model cases of regeneration, which we argue, bridge two distinct stages along a regeneration continuum, encompassing four such stages (Figure 1; the two stages representing the downstream limited regenerative capacities in metazoans are not discussed here). At one end of this regeneration continuum lays WBR of botryllid ascidians (Type 1, the allegory of “stars” in the American flag) and downstream at the regeneration continuum lays the murine DTR (Type 2, the allegory of “strips” in the American flag). T1 and T2 represent two extreme strategies for metazoan regeneration (Table 1; Figure 1). Most model organisms for regeneration are located between both extremes. For example, the *Planaria* regeneration is staged downstream of *B. leachi* on the regeneration continuum, but far upstream of the murine digit tip regeneration. For ranking regeneration events along this continuum, we assigned 15 characteristics that distinguish between T1 and T2 strategies; those involving specific regeneration features (Nos. 1–10 in Table 1) and those operating on biological features at the whole-organism level (Nos. 11–15 in Table 1).

Other fundamental regeneration strategies, assigned to less extensive rehabilitation scenarios, are envisaged downstream to T2 along this regeneration continuum (Figure 1). At the lower end of the regenerative capacity are organisms like *Caenorhabditis elegans* (Figure 1; also so many other nematodes, leeches and birds) that cannot regenerate lost parts (although capable of limited axonal regeneration; [69]) but only capable of tissue homeostasis.

Some important general morphological and biological attributes distinguish regeneration T1 from T2 (Table 1). Hallmarks include the morphological archetypes (coloniality in T1 regeneration versus individuality in T2) and the capacity for asexual reproduction. Furthermore, the botryllid ascidians system corresponds to the extreme case of T1 regeneration, wherein all soma and gonads are regenerated on a weekly basis, through cycles of development termed blastogenesis [21,70,71,72]. T1 regenerating entities grow through somatic embryogenesis and are capable of regenerating germ cells at any ontogenic phase from birth to death. T2 regenerating entities reveal preformistic development, a feature that tags organisms whose entire repertoire of germ cell lineages is irreversibly determined early in ontogeny. According to the latter classification, plants, fungi, multicellular protists and many animals, including placozoans, sponges, cnidarians, platyhelminths, nemerteans, entoproctans, ectoproctans, annelids, hemichordates and urochordates are capable of somatic embryogenesis [54,55,73]. Many of them show T1 regeneration features and others are depleted of super-regeneration power features. Additionally, T1 regenerating metazoans may express pluripotent stemness signatures in both soma and germ cells, possibly reflecting their broad developmental potential for generating diverse cell types. This is an important distinction between T1 and T2 (Table 1). One example of a stemness signature is the Vasa protein, a gene product that is not restricted to the germ cell lineage and is expressed in botryllid ascidians (as in other Animalia) in pluripotent, multipotent, or even in specific differentiated somatic cells [74,75]. With the advent of an exclusively dedicated germ cell lineage in phylogenetically higher evolved organisms, Vasa has become specialized as a germ line specific marker [75]. It is also interesting to note that most soma-expressing Vasa^+^ tissues belong to invertebrate species that have specialized in asexual reproduction (*i.e.*, Hydrozoa, Platyhelminthes, annelids, botryllid ascidians), all of which portray a somatic-embryogenesis type of development. In species with somatic embryogenesis (sponges, *Hydra*, *Planaria*, colonial tunicates) capable of developing germ cells from certain tissues at any ontogenic phase, Vasa expressions are detected not only in the germ lineage but also in somatic cells like neoblasts and interstitial cells that are capable of differentiating into germ cells. In species employing the preformistic mode of development (like the mouse, *C. elegans and D. melanogaster*) very few capacities for regeneration or asexual reproduction are retained and the formation of germ line precursors depends on restricted Vasa expression in germ plasm or its equivalent.

Collecting additional information on the universal biological features dictating regeneration (Table 1) and adding more ubiquitous features to the list will serve us in supporting or refuting the “stars and stripes” paradigm for animal regeneration. Furthermore, analyzing variations on the cellular and molecular levels, among closely related taxa that differ in regeneration powers (e.g., [14,23,44,67,68,76]) or between disparate phyla representing similar regeneration patterns [1,4,23,76], may serve as most powerful tools for elucidating the nature of regeneration. However, the “stars and stripes” paradigm offers a global and inclusive approach to investigating regeneration phenomena based on experimental and analytical tactics. It emerges from “top-down” inquiry, where scrutiny of the system and its features is performed on a global-scale paradigm design. The paradigm emerges from the “bottom-up” reductionist approach that focuses on the regeneration components (cells, molecules), aiming to merge the resulting observations into a paradigm. Independently of the level of detail, paradigms for regeneration will always entail simplifications of specific assumptions, but be useful enough to facilitate experimental works.

## 7. Conclusion

As specified, the “stars and stripes” paradigm allows various combinations of the biological features assigned to T1 and T2 regeneration strategies, along a continuum and downstream of T2 regeneration (Figure 1). It does not consider any concentration gradient or concentration thresholds (as in the Wolpert’s French flag metaphor [35]), and does not refer to the “epimorphosis” and “morphallaxis” terms. This paradigm does not evaluate regeneration across phyla or across body plans, as body parts (e.g., appendages) diverge between taxa; therefore such a comparison becomes inherently problematic. At this stage of analyses, the “stars and stripes” paradigm ignores cases of regeneration loss [6,23,44] that may obscure biological trajectories. The main advantage of the “stars and stripes” paradigm for animal regeneration is that it allows us to compare the biology and the underlying dynamics of the T1 and T2, as well as other modes of regeneration, through critical determining characteristics (Table 1, Figure 1). This will improve formatting of the continuum connecting T1 and T2 regeneration strategies (and downstream strategies) and evaluate the nature of regeneration processes that vary between closely related taxa or within a single organism; altogether creating a solid novel foundation for regeneration studies in Animalia and regenerative medicine.
